# Regulation of BMP2K in AP2M1-mediated EGFR internalization during the development of gallbladder cancer

**DOI:** 10.1038/s41392-020-00250-3

**Published:** 2020-08-13

**Authors:** Xiaoling Song, Maolan Li, Wenguang Wu, Wei Dang, Yuan Gao, Rui Bian, Runfa Bao, Yunping Hu, Defei Hong, Jun Gu, Yingbin Liu

**Affiliations:** 1 Department of General Surgery and Laboratory of General Surgery, Xinhua Hospital Affiliated to Shanghai Jiao Tong University School of Medicine, 1665 Kongjiang Road, 200092 Shanghai, China; 2 Shanghai Key Laboratory of Biliary Tract Disease Research, Shanghai Jiao Tong University, 200127 Shanghai, China; 3 State Key Laboratory of Oncogenes and Related Genes, Shanghai Jiao Tong University, 160 Pujian Road, 200127 Shanghai, China; 4 Department of Biliary-Pancreatic Surgery, Renji Hospital, School of Medicine, Shanghai Jiao Tong University, 160 Pujian Road, 200127 Shanghai, China; 5 Department of General Surgery, Sir Run Run Shaw Hospital, The Medicine School of Zhejiang University, East Qinchun Road, 310020 Hangzhou, Zhejiang Province China

**Keywords:** Gastrointestinal cancer, Gastrointestinal cancer

**Dear Editor,**

Gallbladder cancer (GBC), the most common malignant tumor of the biliary tract, is a highly invasive form of cancer. Surgical resection is currently the first line approach to effectively treat GBC; however, very few patients have the opportunity to receive radical surgical treatment due to lack of obvious symptoms.^1^ The median survival of patients with GBC is only 12 months and 5-year survival rate is <5%, indicating GBC is extremely poor prognosis. Therefore, it is urgent to identify novel key molecules that can potentially serve as early diagnostic biomarkers and/or therapeutic targets. The current study focused on the potential role of bone morphogenetic protein 2 inducible kinase (BMP2K), a serine/threonine kinase, which was recently identified as clathrin-coated vesicle-associated protein in the development of GBC; the outcome may hold diagnostic and therapeutic promising for clinical practice.

To explore this issue, we first examined both mRNA and protein levels of BMP2K in 47 GBC tumor tissue and adjacent or cholecystitis tissue (Supplementary Fig. 1a–d), BMP2K mRNA and protein expression were decreased in GBC tissue than in adjacent or cholecystitis tissue. Decreased expression of BMP2K was significantly correlated with TNM stage and lymph node metastasis, and BMP2K, lymph node metastasis, and TNM stages are independent prognostic factors of GBC (Supplementary Tables 1–3). Consistent with these data, the patients with lower expression of BMP2K had decreased overall survival compared with higher levels of BMP2K (HR = 0.097, *P* < 0.05, Fig. 1a). As expected, endogenous mRNA/protein levels of BMP2K in GBC cell lines were lower than the level expressed in normal gallbladder epithelial cell HGEpC (Supplementary Fig. 2a, b). Exogenous introduction of BMP2K gene to EH-GB1 and OCUG-1 cells significantly suppressed cell proliferation and migration (Supplementary Fig. 2c–h). Animal study confirmed the inhibitory impact of BMP2K on GBC cells in vivo (Supplementary Fig. 2i, j).All these results indicated that BMP2K could inhibit GBC proliferation and migration in vivo and in vitro.

**Fig. 1:**
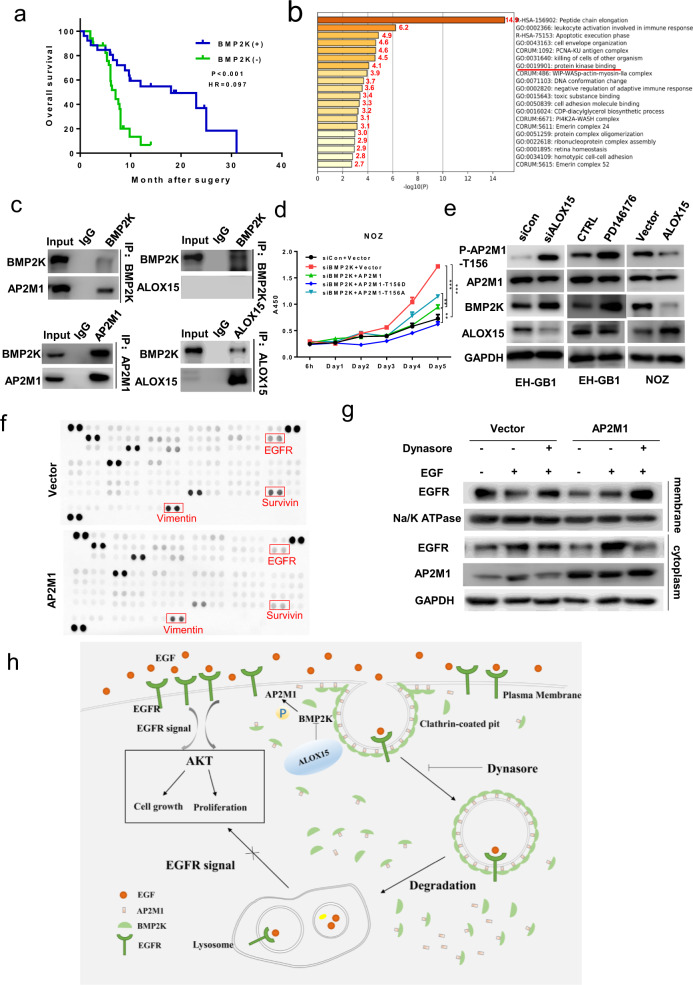
Regulation of BMP2K in AP2M1-mediated EGFR internalization during the development of gallbladder cancer. **a** Kaplan–Meier curve of overall survival among GBC patients stratified based on BMP2K expression. **b** GO analysis of BMP2K and its interaction proteins in Metascape. **c** Co-IP between endogenous BMP2K and AP2M1 or ALOX15. **d** CCK8 analysis of siBMP2Kand AP2M1 or AP2M1-T156A-expressing NOZ cells. **e** Corresponding protein changes in ALOX15-shRNA, overexpressing, or inhibitor-treated cells by western blotting analysis. **f** Human XL Oncology Array Kit from RandD Systems was used to identify the novel mediators in GBC cells. **g** As overexpression AP2M1 treated with EGF or Dynasore (50 mM) for 2 h, membrane surface EGFR was measured by western blotting analysis. **h** A proposed scheme of EGFR internalization regulated by ALOX15–BMP2K–AP2M1 axis

To substantially understand the molecular mechanism of BMP2K underlying inhibition of GBC, we performed immunoprecipitation-mass spectrometry (IP-MS) that can efficiently reveal potential interacting proteins. Consequently, 170 proteins were found as the potential partners of BMP2K (Fig. 1b). To further analyze functional link between BMP2K and its interaction candidates, the Metascape program (http://metascape.org) unveiled 46 proteins involved in protein kinase binding (Fig. 1b). Based on the activity of BMP2K related to clathrin-mediated endocytosis, eight predicted candidate associated with endocytosis (Supplementary Fig. 3a). Combined results of STRING database (https://string-db.org) (Supplementary Fig. 3b), PIK3CG, arachidonate 15‐lipoxygenase (ALOX15), and AP-2 clathrin adaptor complex (AP2M1) were subsequently selected for the following study. We discovered that the endogenous and exogenous BMP2K interacted with AP2M1 and ALOX15 (Fig. 1c, Supplementary Fig. 3c–g), these results support the model that BMP2K interacts with AP2M1 and ALOX15.

Given that protein kinases regulate their substrate activity mainly through protein phosphorylation. And BMP2K is a serine/threonine kinase, we determined the likelihood of serine/threonine phosphorylation of AP2M1 as the substrate of BMP2K. Transfection of Flag-tagged BMP2K led to an increase in serine/threonine phosphorylation of AP2M1 (Supplementary Fig. 4a). Overexpression or downexpression of BMP2K increased or decreased serine/threonine phosphorylation of AP2M1 (Supplementary Fig. 4b). As BMP2K shares 42% amino acid similarity with AAK1 that activates AP2M1 by phosphorylating threonine at 156,^2^ we sought to determine the same activity of BMP2K as AAK1 in AP2M1 phosphorylation. Mutation of AP2M1 at Thr156 led to failure of BMP2K to phosphorylate AP2M1 (Supplementary Fig. 4c), and the western blotting results showed that overexpression or knockdown expression of BMP2K increased or decreased, respectively, the expression of phosphorylated AP2M1 at Thr156, but did not alter the non-phosphorylated AP2M1 (Supplementary Fig. 4d), which indicate that BMP2K induces AP2M1 threonine phosphorylation at 156. Moreover, the results of co-expression of si-BMP2K and AP2M1 showed cell proliferation inhibition of BMP2K is at least partially mediated by AP2M1 phosphorylation at Thr156 (Fig. 1d).

ALOX15 has been implicated in many pathologic aspects of tumor progression including angiogenesis, inflammation, and metastasis.^3^ In this study, we found inhibition of ALOX15 using either knockdown of ALOX15 gene or a specific inhibitor PD146176 suppressed EH-GB1 cell proliferation and overexpression of ALOX15 in NOZ cells promoted cell proliferation (Supplementary Fig. 5a–c). To investigate the relationship between ALOX15 and BMP2K, western blotting assay indicates knockdown of ALOX15 gene induced expression of BMP2K and phosphorylated AP2M1-T156, whereas overexpression of ALOX15 inhibited the expression of BMP2K and phosphorylated AP2M1 at Thr156 (Fig. 1e). In vivo experiment confirmed our assumption that ALOX15, as the upstream of BMP2K, negatively regulates BMP2K (Supplementary Fig. 5d, e).

To interrogate what are potential downstream effectors of AP2M1 that may mediate the development of GBC, Human XL Oncology Array Kit from RandD Systems was used to detect differential downstream protein expression profile of ALOX15/BMP2K/AP2M1. AP2M1 overexpression decreased expression of EGFR and others (Fig. 1f). Nakazawa et al.^4^ reported that overexpression of EGFR has been implicated in GBC progression. It is emerging that degradation and ultimate fate of EGFR protein in the cells determine the duration of its membrane kinase activity. EGFR harbors a YXXΦ consensus sequence in its cytoplasmic tail, which binds to AP2M1 to initiate internalization signaling.^5^ AP2M1 unexpectedly failed to associate with EGFR (Supplementary Fig. 6a), however, overexpression of AP2M1 decreased expression of EGFR and its intracellular signaling p-AKT upon exposure to EGF from 0 to 30 min, overexpression of AP2M1-T156A partially attenuated the decreased levels of EGFR compared with AP2M1 (Supplementary Fig. 6a–c). In addition, AP2M1 overexpression induced EGFR translocation from membrane to cytoplasm, and the translocation induced by AP2M1 was blocked by Dynasore, an inhibitor of endocytic pathways (Fig. 1g). The results of flow cytometry were in agreement with western blotting results, which demonstrate that AP2M1 mediates EGF-induced EGFR internalization through cellular endocytosis (Supplementary Fig. 6d).

In order to provide clinical evidence strongly supporting the potential regulation of EGFR by the ALOX15–BMP2K–AP2M1 axis in GBC, their expression associations in cancer tissues were detected, and the results showed a positive correction between BMP2K and AP2M1, but a negative correlation between BMP2K and ALOX15 (Supplementary Fig. 7a). In concert with these associations, IHC studies indicated 60 GBC expressed higher levels of ALOX15 but lower levels of AP2M1 than those found in cholecystitis tissues (Supplementary Fig. 7b, c). Collectively, these results indicate that ALOX15–BMP2K–AP2M1 axis acts as a key molecular mechanism to regulate EGFR internalization during GBC development (Fig. 1h).

In sum, we found that ALOX15 negatively regulates BMP2K expression that phosphorylates AP2M1 at Thr156 and subsequently induces EGFR internalization. Our data suggest a novel regulatory axis of ALOX15–BMP2K–AP2M1 underlying EGFR endocytosis, underscoring potential therapeutic targets for GBC therapy.

## Supplementary information

Supplementary Information

## Data Availability

All data and materials are available to the researchers once published.

## References

[1] Li M (2014). Whole-exome and targeted gene sequencing of gallbladder carcinoma identifies recurrent mutations in the erbb pathway. Nat. Genet..

[2] Ricotta D, Conner SD, Schmid SL, von Figura K, Honing S (2002). Phosphorylation of the AP2 mu subunit by AAK1 mediates high affinity binding to membrane protein sorting signals. J. Cell Biol..

[3] Kuhn H, Banthiya S, van Leyen K (2015). Mammalian lipoxygenases and their biological relevance. Biochim Biophys. Acta.

[4] Nakazawa K (2005). Amplification and overexpression of c-erbB-2, epidermal growth factor receptor, and c-met in biliary tract cancers. J. Pathol..

[5] Owen DJ, Evans PR (1998). A structural explanation for the recognition of tyrosine-based endocytotic signals. Science.

