# Supplementary material for: Regulation of BMP2K in AP2M1-mediated EGFR internalization during the development of gallbladder cancer
Source: Signal Transduct Target Ther. 2020 Aug 13;5:154. doi: 10.1038/s41392-020-00250-3 (PMC7426970; doi:10.1038/s41392-020-00250-3)
Supplement: Supplementary file 1 — Supplementary Information [file 41392_2020_250_MOESM1_ESM.docx]

**Regulation of** **BMP2K in AP2M1-mediated EGFR internalization during the development of gallbladder cancer**

Xiaoling Song^#134^, Maolan Li^#13^,Wenguang Wu^#123^, Wei Dang^#134^, Yuan Gao^1234^,Rui Bian^1234^,Runfa Bao^134^, Yunping Hu^1234^,Defei Hong*^5^, Jun Gu*^13^, Yingbin Liu*^1234^

#These authors contributed equally to this work.

*To whom correspondence should be addressed:

Yingbin Liu(Tel:+86-021-25078813, Fax:+86-021-65030840;Email: liuyingbin@xinhuamed.com.cn);Jun Gu (Email: gujun02@xinhuamed.com.cn);

Defei Hong (Email: hongdefei@163.com)

Supplemental information contains online methods, 8 supplemental figure and legend, and 1 tables.

**Materials and Methods**

**Cell culture and Reagents**

GBC-SD cell line from the Cell Bank of the Chinese Academy of Sciences (Shanghai, China), and NOZ, SGC-996, EH-GB1 and OCUG-1 cell lines from the Health Science Research Resources Bank (Osaka, Japan). The gallbladder epithelial cell HGEpC was established and characterized by the Shanghai key laboratory of biliary tract disease research. NOZ cells were cultured in William's medium (Gibco, Grand Island, NY, USA), GBC-SD, OCUG-1 and EH-GB1 cells were cultured in DMEM (Gibco); and SGC-996 cells were cultured in RPMI 1640 medium (Gibco). All cells were supplemented with 10% fetal bovine serum and 100 μg/ml penicillin/streptomycin.

**RNA oligonucleotides and plasmids transfection assays**

Small Interfering RNAs (siRNA) were synthesized by Biotend Biotechnology Company (Shanghai, China), and short hairpin shRNA were synthesized by GenePharma Company (Shanghai, China). The sequences of siRNAs were shown in Table S1. The plasmids were constructed by Lqbiotech (Shanghai, China). Cells were seeded on 6-well plates and transfected for 48h using a transfection reagent, according to the manufacturer's protocol.

**CCK-8 assay and colony formation assay**

Cell proliferation was assessed with the CCK-8 assay following the manufacturer’s instructions. Cells were seeded in 96-well plates at the density of 1000 cells/well, CCK-8 (10 μl) was added to each well, and incubated for 2h. The cell proliferation curves were plotted after assessing absorbance at 450 nm.

400 cells/well were individually seeded into 6-well plates and incubated for 2 weeks to allow colony formation. The culture medium was changed every three days. After that, colonies were fixed with 4% paraformaldehyde and stained with 0.1% crystal violet for 30 min. Then the colonies (with more than 50 cells) were observed under a microscope.

**Immunoprecipitation-Mass spectrometry(****IP-MS) assay**

Protein samples were loaded onto a 10% acrylamide gel (ThermoFisher Scientific, Waltham, MA). After briefly running the gel, sample lanes were excised. Proteins ingel were digested with trypsin and analyzed by MS-MS at the Shanghai Applied Protein Technology.

**Co-****Immunoprecipitation(****Co-IP) and Western blotting**

Cells were lysed in NETN buffer (150 mM NaCl, 50 mM Tris, and 0.3% NP-40 [pH 7.5]) with protease inhibitors (Biotool, catalog number B14001) at 4°C for 30 min. The cell lysates were incubated with 2 μg of primary antibody against the target protein for 4 h at 4°C. Then 30 μl protein A/G Plus agarose beads (Santa Cruz, Biotechnology) were added to the cell lysates and incubated at 4°C overnight. The immunoprecipitates were collected by centrifugation at 5000 rpm for 5 min at 4°C. Then the beads were washed at least three times in NETN buffer and boiled in 2× SDS loading buffer to dissociate the protein from the beads. Standard western blotting protocols were used to analyze the results of the IP experiments.

For western blotting, total cell lysates were separated on a 10% SDS-PAGE gel and transferred electrophoretically to PFDV membrane(Millipore, Bedford, MA, USA) at 100 V for 100 minutes. The blots were blocked in 5% milk for 1 hour, and then incubated with specific primary antibodies at 4°C overnight. Membranes were then incubated with corresponding secondary antibodies for 1 hour and visualized using the enhanced chemiluminescent detection reagent.

**RNA extraction and** **qRT-PCR**

Total RNA was extracted with TRIzol reagent(Invitrogen, Carlsbad, CA, USA) according to the manufacturer’s instructions. cDNA was amplified by qRT-PCR with a SYBR-Green method (TaKaRa) and the primer sequences were shown in Table S2. The expression of target genes was normalized to expression of the housekeeping gene GAPDH.

**Immunofluorescence(****IF) assay**

GBC cells were fixed with 4 % paraformaldehyde for 20 min, permeabilized in 0.2 % Triton for 10 min and blocked in 1%BSA for 1 h at room temperature. The cells were incubated with primary antibodies at 4 °C overnight, followed by staining with Alexa Fluor 488 and Cy3-conjugated secondary antibodies for 1 h at room temperature. Coverslips were counter-stained with DAPI (1 ng/mL) to visualize the nuclei. After staining, the cells were thoroughly washed with PBS and imaged with a confocal laser scanning microscopy (Leica, Buffalo Grove, IL).

**In vitro** **phosphorylation assay**

In vitro phosphorylation assay was performed as previously described[1-2]. Briefly, the indicated plasmids were separately transfected into GBC cells and the cells were lysed using NETN buffer. Transfected cells were immunoprecipitated using anti-phospho-(ser/thr)Phe antibody and protein G-agarose. The phosphorylation levels were examined by western blotting assay.

**EGFR internalization analysis**

Cells were washed with ice‐cold PBS and detached by trypsin on ice. Then the cells were fixed 10 min in ice‐cold 2% paraformaldehyde. The amount of EGFR present at the cell surface was determined by labeling of the cells with an anti‐EGFR antibody or anti‐EGFR IgG2A antibody followed by flow cytometry to quantify EGFR surface labeling.

**Clinical specimens and** **immunohistochemistry(****IHC) staining**

The study was approved by the ethics committee of Xinhua Hospital, and all patients provided informed consent(Approval No.XHEC-D-2018-076). GBC tissue specimens were obtained from 60 patients who underwent radical cholecystectomy (without prior radiotherapy or chemotherapy) between 2014 and 2016 at the Department of General Surgery, Xinhua Hospital, Shanghai Jiao Tong University School of Medicine, China. Diagnosis of GBC, and cholelithiasis, the presence of lymph node metastases, and tumor differentiation were confirmed by hematoxylin and eosin (H&E) staining. IHC was performed according to previously described methods[3]. Samples were semi-quantitatively scored for the percentage of cells with immunoreactions as follows: 0,0% immunoreactive cells; 1,<10% immunoreactive cells; 2,11–50% immunoreactive cells and 3,>50% immunoreactive cells. Additionally, the staining intensity was scored as 0 (negative), 1 (weak), 2 (moderate), or 3 (strong).The final immunoreaction score was defined as the sum of both parameters (extension and intensity), and the samples were classified as negative (0), weakly stained (1–2), moderately stained (3), and strongly stained (4–6). For statistical purposes, only moderate and strong final immunoreaction scores were considered positive; the other final scores were considered negative.

**Animal Studies**

All mice were housed under specific pathogen-free conditions following the guidelines of the Ethics Committee of Xinhua Hospital, School of Medicine, Shanghai Jiaotong University (Approval No.XHEC-F-2018-024) . 4- to 6-week-old nu/nu nude male mice were purchased from the Shanghai Laboratory Animal Centre of the Chinese Academy of Sciences (Shanghai, China).The mice were randomly divided into the indicated groups (5 mice/group) before inoculation. 5 × 10^6^ transfected GBC-SD or NOZ cells were injected into the left flank of the mice. Tumor volume was calculated using the formula width^2^ (mm^2^)×length (mm)/2, where width and length were the shortest and longest diameters, respectively. After approximately 4 weeks, mice were sacrificed, and primary tumors were collected for further immunohistochemical staining and western blot analysis

**Statistical analysis**

All data are expressed as the mean ± SD. Statistical differences between 2 groups were evaluated using a 2-tailed t test. Multiple group comparisons were performed using a 1-way ANOVA followed by Dunnett’s post-hoctest. The significance of a Kaplan-Meier survival plot was determined by log-rank analysis. Statistical analysis was performed using GraphPad Prism 6 and SPSS 22.0 software. A P value of less than 0.05 was considered statistically significant.

Reference:

. Janjanam J, Chandaka GK, Kotla S, Rao GN. PLCβ3 mediates cortactin interaction with WAVE2 in MCP1-induced actin polymerization and cell migration. *Mol Biol Cell*. **26**,4589-606(2015).

2. Xu Q, et al. HSP90 promotes cell glycolysis, proliferation and inhibits apoptosis by regulating PKM2 abundance via Thr-328 phosphorylation in hepatocellular carcinoma. *Mole Cancer.* **16**,178(2017).

3.Hu YP, et al. STYK1 promotes cancer cell proliferation and malignant transformation by activating PI3K-AKT pathway in gallbladder carcinoma. *Int J Biochem Cell Biol.* **97**,16-27(2018).

| **Table S1. Association between BMP2K expression with the clinicopathological parameters of GBC.** | | | | | | | |  |
| --- | --- | --- | --- | --- | --- | --- | --- | --- |
| **Parameter** | **Category** | | **No. of cases** | **BMP2K expression** | | | |  |
|  |  |  |  | **No. of positive cases (%)** | | ***χ^2^*** | ***P* value** |  |
| Age | <60 | | 29 | 10(38.5) | | 1.791 | 0.181 |  |
|  | ≥60 | | 31 | 16(61.5) | |  |  |  |
| Sex | male | | 25 | 9(34.5) | | 0.939 | 0.333 |  |
|  | female | | 35 | 17(65.4) | |  |  |  |
| Histopathological Subtypes | high | | 15 | 8(30.8) | | 1.056 | 0.590 |  |
|  | middle | | 27 | 10(38.5) | |  |  |  |
|  | low | | 18 | 8(30.8) | |  |  |  |
| TNM Stage | 1-II | | 30 | 18(69.2) | | 6.787 | 0.009* |  |
|  | III-IV | | 30 | 8(30.8) | |  |  |  |
| Lymph Node Metastasis | Negative | | 25 | 17(65.4) | | 10.619 | <0.001* |  |
|  | Positive | | 35 | 9(34.6) | |  |  |  |
|  | | | |  | |  |  |  |
|  | |  | | |  | | | |

| **Table S2: Univariate log-rank analysis of overall survival (OS)** | | | | | |
| --- | --- | --- | --- | --- | --- |
| **Parameter** | **Category** | **Case number** | **Median survival time(month)95%CI** | **Hazard ratio** | **p value** |
| Age | <60 | 29 | 8.000(6.426-9.574) | 1.21 | 0.553 |
|  | ≥60 | 31 | 8.000(6.182-9.818) |  |  |
| Sex | male | 25 | 6.100(5.197-7.003) | 1.387 | 0.31 |
|  | female | 35 | 8.000（6.895-9.105) |  |  |
| Histopathological Subtypes | high | 15 | 9.800(5.273-8.727) | 0.925 | 0.842 |
|  | middle | 27 | 8.000(7.031-8.968) |  |  |
|  | low | 18 | 7.000(5.273-8.727) |  |  |
| TNM Stage | 1-II | 30 | 18.000(7.755-28.245) | 8.985 | ＜0.01 |
|  | III-IV | 30 | 6.000(5.818-6.182) |  |  |
| Lymph Node Metastasis | Negative | 25 | 23.000(7.311-38.689) | 14.226 | ＜0.01 |
|  | Positive | 35 | 6.000(5.792-6.248) |  |  |
| BMP2K Expression | Negative | 34 | 7.000（5.539-8.461） | 0.097 | ＜0.01 |
|  | Positive | 26 | 6.219（5.881-30.189） |  |  |

| **Table S3:Multivariate analysis of overall survival (OS)** | | | | |
| --- | --- | --- | --- | --- |
| **Parameter** | **Category** | **Hazard ratio** | **95%CI** | **P value** |
| Age | <60 | 1.009 | 0.513-1.985 | 0.98 |
|  | ≥60 |  |  |  |
| Sex | male | 1.44 | 0..710-2.922 | 0.312 |
|  | female |  |  |  |
| Histopathological Subtypes | high | 0.785 | 0.493-1.165 | 0.206 |
|  | middle |  |  |  |
|  | low |  |  |  |
| TNM Stage | 1-II | 3.41 | 1.317-8.826 | 0.011 |
|  | III-IV |  |  |  |
| Lymph Node Metastasis | Negative | 5.205 | 1.453-17.385 | 0.011 |
|  | Positive |  |  |  |
| BMP2K Expression | Negative | 0.202 | 0.066-0.616 | 0.005 |
|  | Positive |  |  |  |


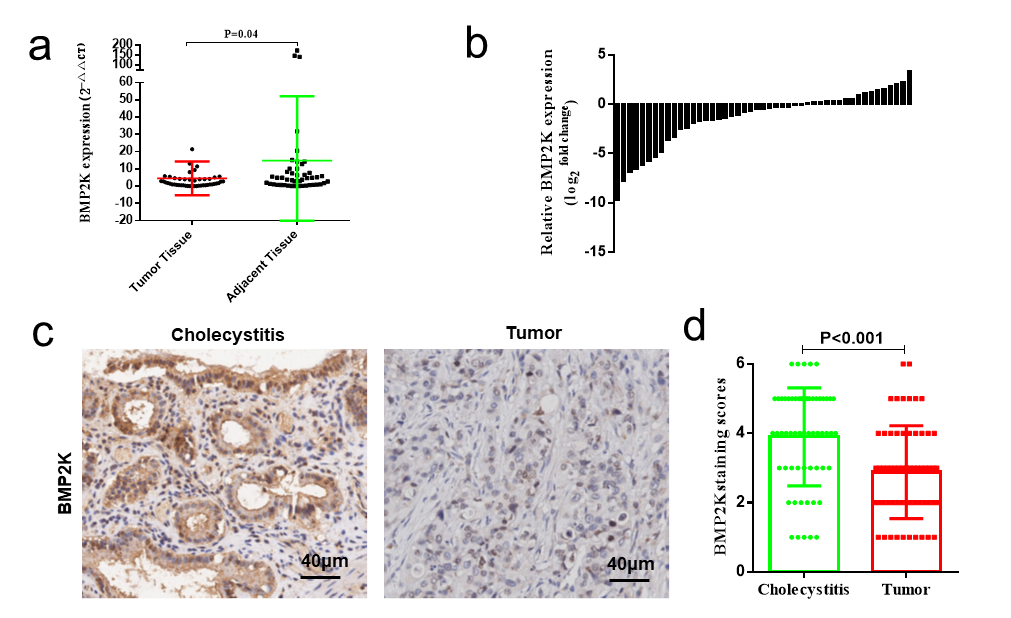


**Figure S1** **BMP2K is down-regulated in GBC** a-b) qRT-PCR analysis of BMP2K expression in 47 pairs of GBC tumor tissues and adjacent tissues. c)Representative IHC staining images of GBC and cholecystitis patient samples. d)Scatterplots of the average staining scores of BMP2K expression in patients with GBC or cholecystitis.


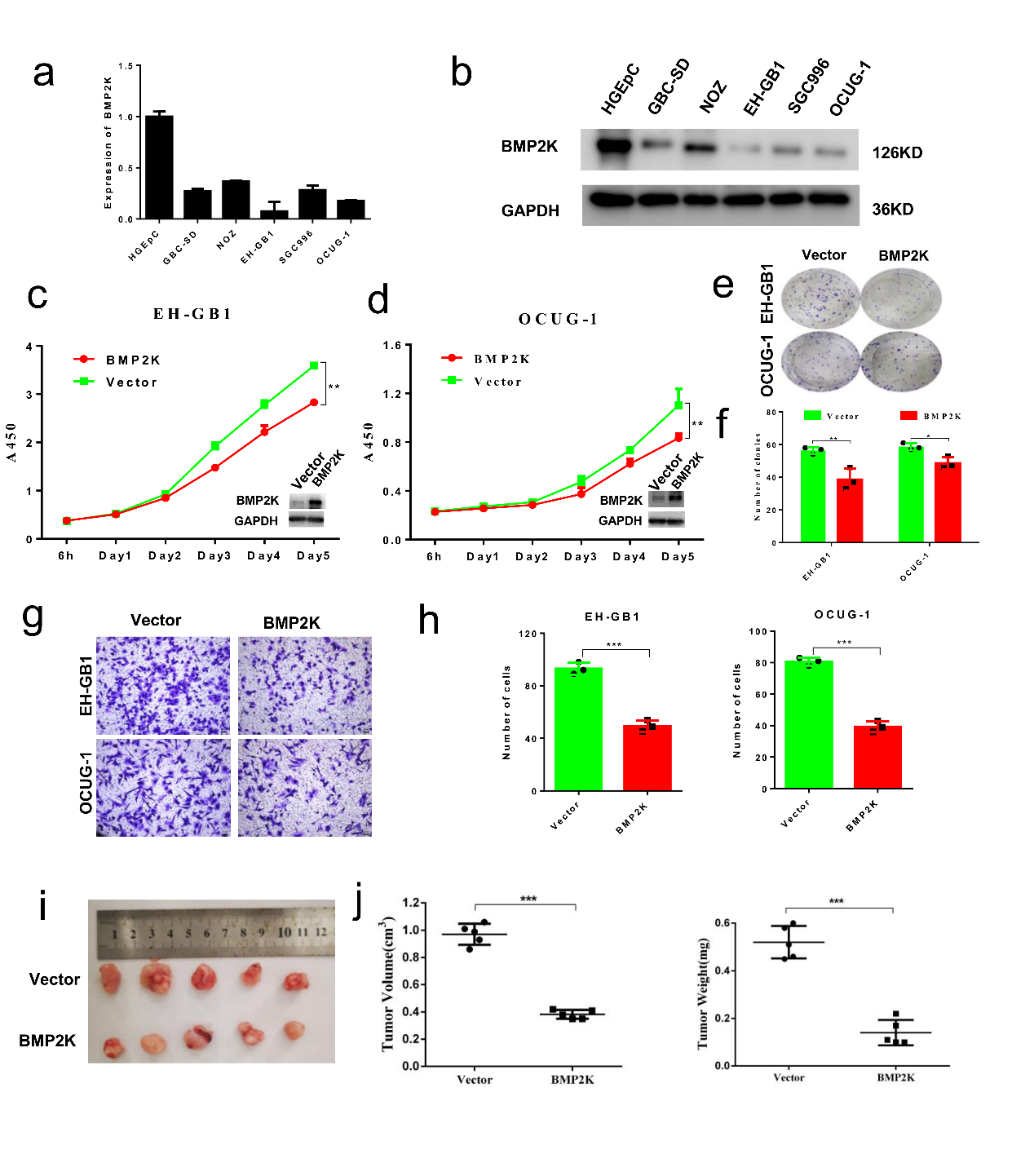
**FigureS2 BMP2K inhibits GBC cells growth *in vitro* and *in vivo*** a-b)qRT-PCR analysis and western blotting of BMP2K expression in GBC cells and normal gallbladder epithelium cells. c-d) CCK8 assay was performed to analyze the effects of BMP2K on GBC cell proliferation. e-f) Colony formation assay was performed in GBC cells, and the number of colonies were determined and statistically analyzed. g-h) Transwell assay was conducted in BMP2K-overexpression cells. i)BMP2K-overexpression or control EH-GB1 cells were injected subcutaneously into nude mice. Images of 5 representative mice from each group were presented. j)Tumor volume and weight were measured from the animals.*P < 0.05, **P < 0.01, ***P < 0.001.


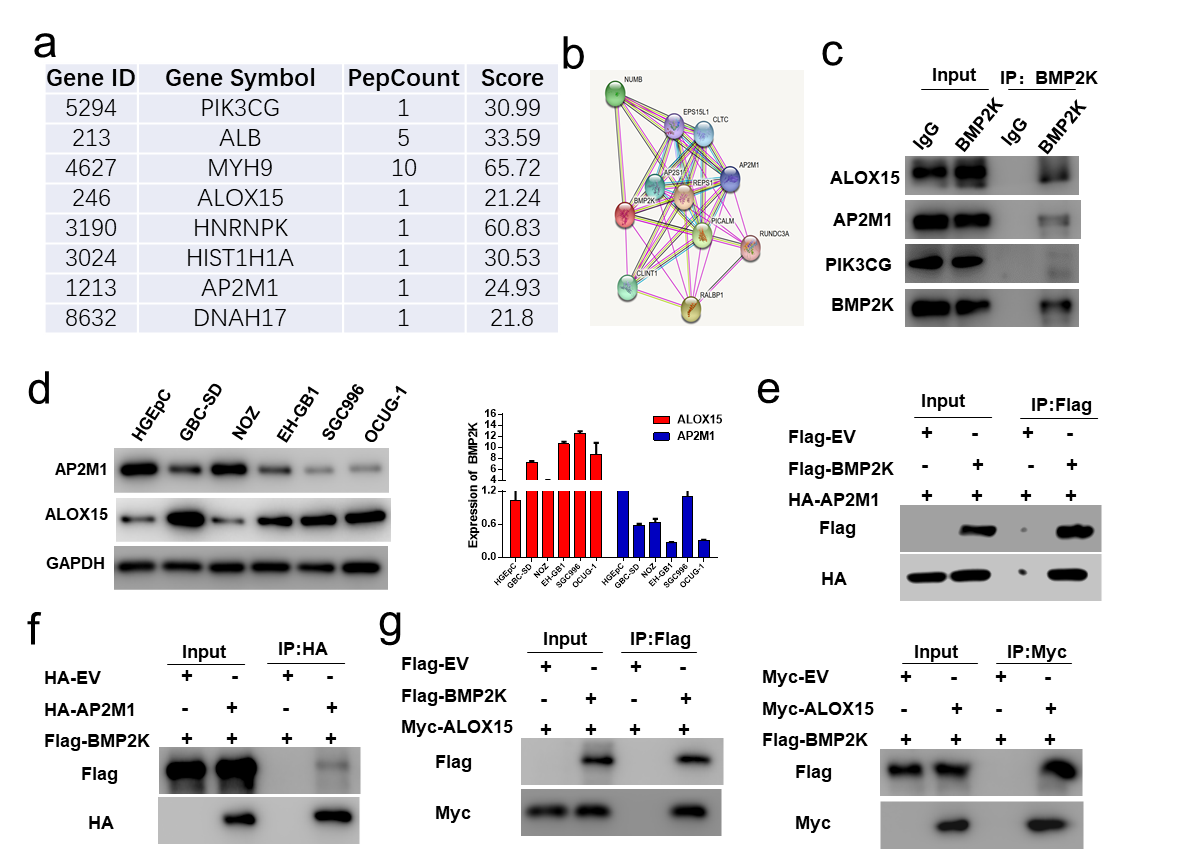


**FigureS3 BMP2K-interacting proteins in GBC cells** a) Proteins related to endocytosis were predicted in Metascape. b)Protein–protein interaction network of BMP2K in the STRING dataset. c) Co-IP analysis between BMP2K and its potential interaction proteins. d) Western blotting and qPCR assay of ALOX15 and AP2M1 in GBC cells and normal gallbladder epithelium cells. e-g) Co-IP between exogenous BMP2K and AP2M1 or ALOX15.


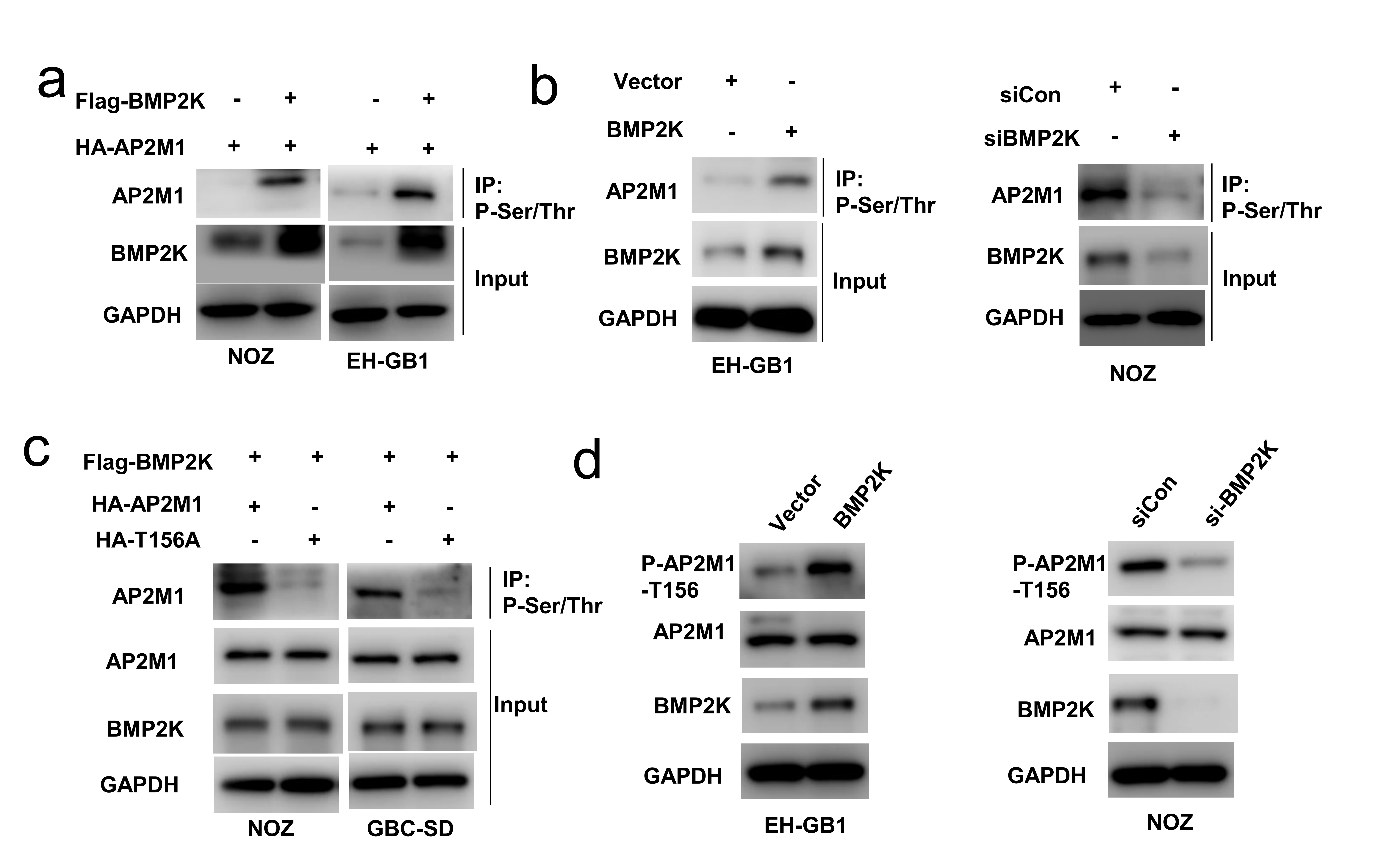


**FigureS4 BMP2K induces AP2M1 phosphorylation at Thr156.** a)Flag-BMP2K and HA-AP2M1 plasmids were transfected into GBC cells. Immunoprecipitation was performed using an anti-Flag or anti-HA antibody. Representative IP experiments were performed to examine BMP2K Ser/Thr phosphorylation. b) Representative IP experiments were performed to examine BMP2K Ser/Thr phosphorylation in GBC cells. c)Representative IP experiments were performed to examine BMP2K Ser/Thr phosphorylation in GBC cells transfected with Flag BMP2K, together with HA-AP2M1 or AP2M1-T156A mutant. d) Expression of P-AP2M1-T156 in GBC cells overexpressing BMP2K or shRNA BMP2K.


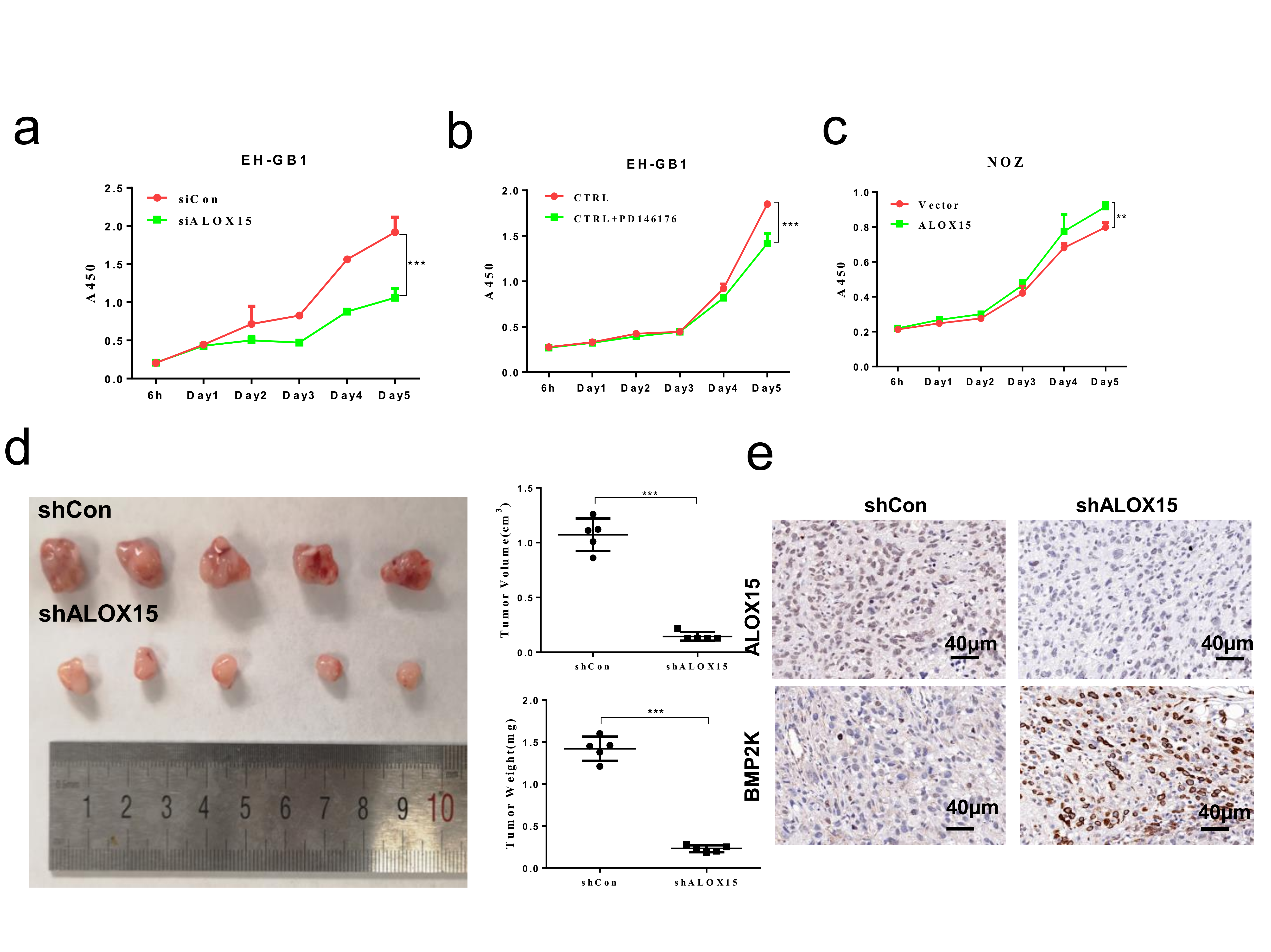


**FigureS5 ALOX15 inhibits the expression of BMP2K** a-b)CCK8 analysis of ALOX15-knockdown cells. c)CCK8 analysis of ALOX15-overexpressing cells. d)ALOX15-knockdown or control cells were injected subcutaneously into nude mice. Images of 5 representative mice from each group were presented. Tumor volume and weight were measured from the animals. e) ALOX15 and BMP2K expression of the animals by IHC assay.*P < 0.05, **P < 0.01, ***P < 0.001.


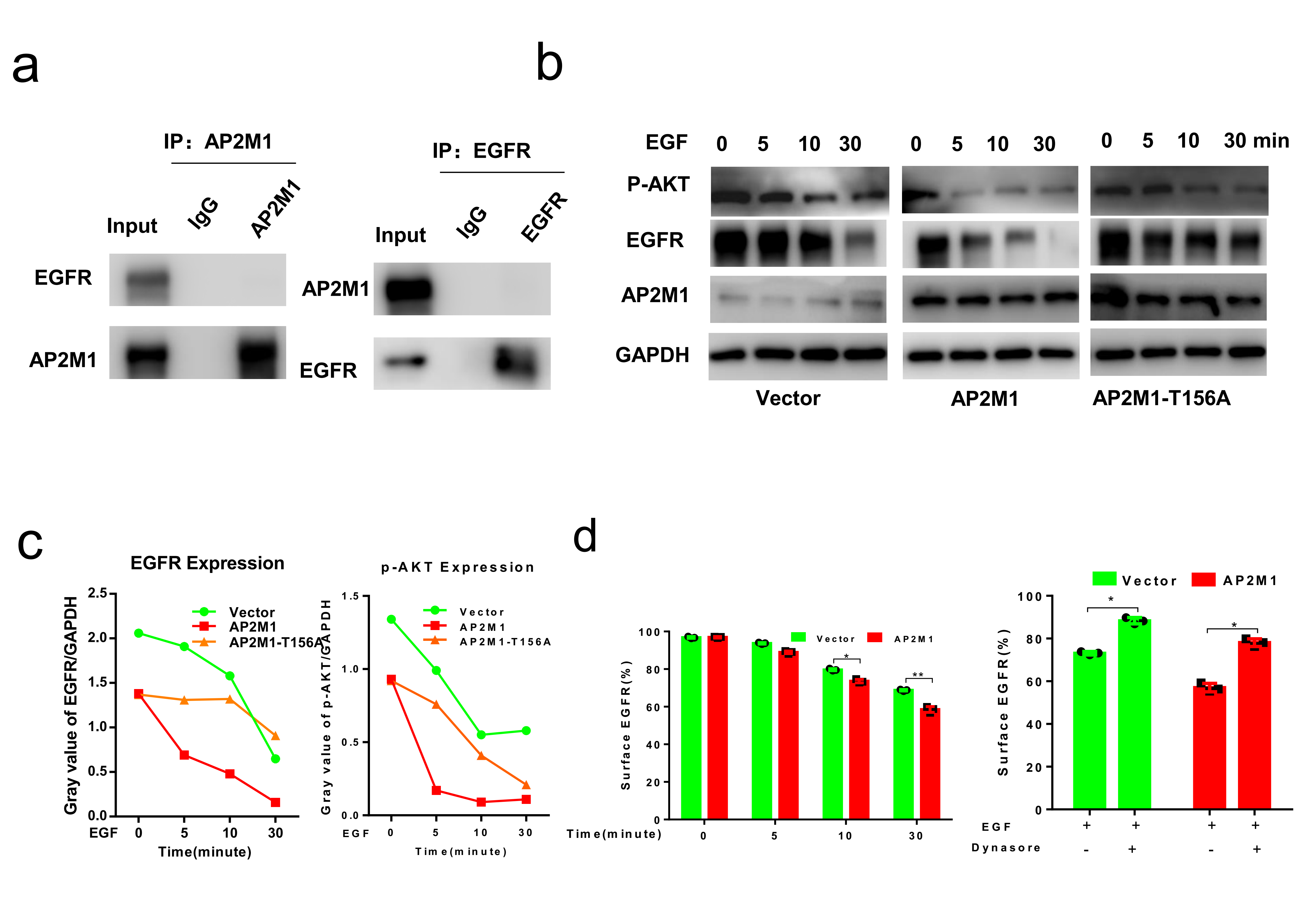


**FigureS6 AP2M1 induces EGFR internalization.** a) Co-IP between endogenous EGFR and AP2M1. b-c)Overexpressed AP2M1 or AP2M1-T156A cells were stimulated with EGF (20 ng/ml) for different time points. EGFR and p-AKT were determined by western blotting analysis. d) AP2M1-overexpressing cells treated with EGF or Dynasore as above, membrane surface EGFR was measured by flowcytometry.

**
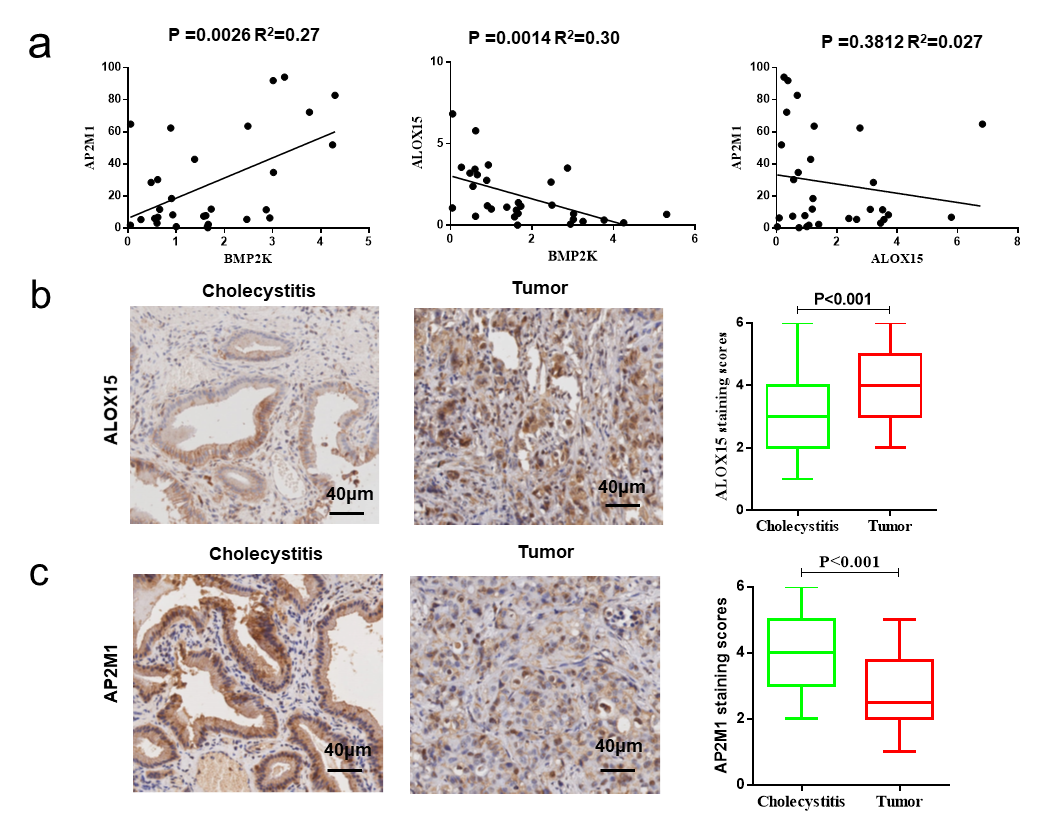
**

**FigureS7 The clinicopathologic relationship of ALOX15-BMP2K-AP2M1 axis.** a) The correlations of ALOX15, BMP2K, andAP2M1in 60 GBC cancers. b-c) Representative IHC staining images of ALOX15 and AP2M1 in GBC and cholecystitis patient samples. *P < 0.05, **P < 0.01, ***P < 0.001.f) A proposed scheme of EGFR internalization regulated by ALOX15-BMP2K-AP2M1 axis.
